# Predictive models for thromboembolic events in giant cell arteritis: A US veterans health administration population-based study

**DOI:** 10.3389/fimmu.2022.997347

**Published:** 2022-11-09

**Authors:** Despina Michailidou, Tianyu Zhang, Nicole M. Kuderer, Gary H. Lyman, Andreas P. Diamantopoulos, Pavlos Stamatis, Bernard Ng

**Affiliations:** ^1^ Division of Rheumatology, Department of Medicine, University of Washington, Seattle, WA, United States; ^2^ Rheumatology Section, Veterans Affairs (VA) Puget Sound Healthcare System, Seattle, WA, United States; ^3^ Department of Biostatistics, University of Washington, Seattle, WA, United States; ^4^ Advanced Cancer Research Group, Kirkland, WA, United States; ^5^ Public Health Sciences and Clinical Research Divisions, Fred Hutchinson Cancer Center, Seattle, WA, United States; ^6^ Schools of Medicine, Public Health and Pharmacy, University of Washington, Seattle, WA, United States; ^7^ Department of Rheumatology, Martina Hansens Hospital, Oslo, Norway; ^8^ Department of Clinical Sciences Lund, Rheumatology, Lund University, Lund, Sweden

**Keywords:** giant cell arteritis, thromboinflammation, pulmonary embolism, deep venous thrombosis, thromboembolic events, nomograms, thrombocytosis, predictors

## Abstract

Giant cell arteritis (GCA) that affects older patients is an independent risk factor for thromboembolic events. The objective of this study was to identify predictive factors for thromboembolic events in patients with GCA and develop quantitative predictive tools (prognostic nomograms) for pulmonary embolism (PE) and deep venous thrombosis (DVT). A total of 13,029 patients with a GCA diagnosis were included in this retrospective study. We investigated potential predictors of PE and DVT using univariable and multivariable Cox regression models. Nomograms were then constructed based on the results of our Cox models. We also assessed the accuracy and predictive ability of our models by using calibration curves and cross-validation concordance index. Age, inpatient status at the time of initial diagnosis of GCA, number of admissions before diagnosis of GCA, and Charlson comorbidity index were each found to be independent predictive factors of thromboembolic events. Prognostic nomograms were then prepared based on these predictors with promising prognostic ability. The probability of developing thromboembolic events over an observation period of 5 years was estimated by with time-to-event analysis using the method of Kaplan and Meier, after stratifying patients based on predicted risk. The concordance index of the time-to-event analysis for both PE and DVT was > 0.61, indicating a good predictive performance. The proposed nomograms, based on specific predictive factors, can accurately estimate the probability of developing PE or DVT among patients with GCA.

## Introduction

Giant cell arteritis (GCA), a form of large vessel vasculitis, is a chronic systemic inflammatory disease of unknown etiology that primarily affects older patients (1). GCA can be complicated by thromboembolic events such as pulmonary embolism (PE) and deep venous thrombosis (DVT) (2).

In recent years, terms such as immunothrombosis and thromboinflammation have been emerged. In the absence of infection, uncontrolled inflammation may induce a pro-coagulant state leading to the development of thrombotic events (3, 4). Recent studies demonstrate that patients with GCA have a higher incidence of PE and DVT within the first year of their diagnosis (5) and an increased risk of cerebrovascular events (6), especially in the presence of atherosclerotic risk factors at the time of diagnosis of GCA (7). Atherosclerotic risk factors are also known to be significantly associated with venous thrombotic events (8). In another report, an increased risk of thromboembolism was related to the period of higher disease activity at the onset of GCA (9). However, in a different study the incidence of both thromboembolic and cerebrovascular events did not differ between biopsy confirmed GCA and non-GCA patients (10). Interestingly, hospitalizations in patients with GCA were found to increase the risk of thromboembolic events compared to hospitalizations in the general population (11).

It is also known that thrombocytosis is associated with GCA (12). Leukocyte and platelet activation may contribute to vessel inflammation and possibly to thromboembolic events in patients with GCA (13). In a small study, 13 out of 19 patients with biopsy proven GCA had evidence of thrombocytosis several months to a year before the diagnosis and treatment of GCA (14). Medications that can be “thrombogenic” include diuretics that may cause hemoconcentration and lead to increased blood viscosity *via* changes in fibrinogen concentrations and coagulation time (15). Of note a recent study demonstrated that diuretic use might be weakly associated with a lower risk of GCA (16).

Predictive factors for thromboembolic events in patients with GCA have not been carefully investigated. To the best of our knowledge, there are no models that provide quantitative prediction of thromboembolic events among patients diagnosed with GCA. One of the main objectives of this study was to propose specific predictive factors that will help physicians to better assess patient risk for thromboembolic events and develop quantitative tools (such as nomograms) for prediction in patients with a diagnosis of GCA among a Veteran-based population. The secondary objective of our study was to perform a time-to-event analysis to estimate the probability of a thromboembolic event over an observation period of 5 years.

## Methods

### Data sources and study population

In this institutional review board (IRB)-approved retrospective study (#01854) patients with GCA were included between January 1^st^ 1999 and January 13^th^ 2022. The Veterans Affairs Clinical Data Warehouse was used for data extraction and collection. Thromboembolic events were identified, by diagnostic codes of hospital admissions and outpatient visits within the Veterans Health Administration system as previously conducted by our group (5).

### Study design and data collection

Our study cohort included patients with a diagnosis of GCA without a history or prior diagnosis of GCA over the prior 2 years. The study cohort included patients: (a) aged ≥50 years; and (b) classified with at least one GCA clinical modification code from the International Classification of Diseases Ninth or Tenth Revision (ICD-9-CM or ICD-10-CM) (**Supplementary Table 1**) by a rheumatologist or other health provider.

The following data were collected: patient age, race/ethnicity, sex, baseline body mass index (BMI), smoking status prior to the diagnosis of GCA, use of aspirin, anticoagulants (heparin, warfarin, low molecular weight heparin, rivaroxaban, apixaban) or diuretics (thiazide, loop, potassium sparing, and osmotic diuretics, carbonic anhydrase inhibitors) within 5 years prior to diagnosis of GCA. The Charlson comorbidity index, which is frequently used as a measure of general health based on 19 medical conditions (17, 18), was also generated using data up to 5 years prior to the index date. Thrombocytosis was defined as platelet count ≥ 450x10^9^/L (19) between a two-week period before and after the date of diagnosis of GCA. Data about the initial diagnosis of GCA in the outpatient or inpatient setting, and number of hospital admissions within 5-years for any cause before diagnosis of GCA, were also recorded. These clinical and laboratory covariates were then used as potential predictive factors.

### Study outcomes of interest

The main outcomes of interest were the incidence of PE or DVT after the diagnosis of GCA. We defined PE and DVT events using ICD‐9 and ICD-10 procedure codes (**Supplementary Table 1**).

### Statistical analysis

Baseline demographic characteristics were detailed based on the stratification with or without thromboembolic events after the diagnosis of GCA. We reported mean ± standard deviation for quantitative variables. For categorical variables counts and proportions (based on all non-missing observations) are presented. Covariates specified *a priori* age, race, sex, BMI, smoking status, Charlson comorbidity index, diagnosis of GCA in the outpatient versus inpatient setting, number of hospital admissions for any reason before diagnosis of GCA, use of anticoagulation, aspirin and diuretics as well as hypertension within 5 years prior to the diagnosis of GCA, and thrombocytosis to be assessed as potential predictors in our study.

Univariable and multivariable Cox proportional hazards regression models were then applied to explore potential predictors of PE and DVT events for patients with GCA. The censoring events in the time-to-event analysis were: 1) death, 2) end of study (January 13^th^ 2022), and 3) development of malignancies. The baseline covariates we used for these models are reported in **Table 1**. We report the hazard ratio (HR) with 95% confidence interval (CI) for each potential predictive factor, together with the associated p-value in **Tables 2**, **3**. For all our analyses, p-values < 0.05, were considered statistically significant.

**Table 1:** Baseline demographic characteristics of GCA patients without and with thromboembolic events.

Groups	GCA without thromboembolic events	GCA with thromboembolic events (VTE)
N	12403	626
Sex (male), N (%)	11,634 (94)	597 (95)
Race/Ethnicity, N (%)		
Native American	101 (1)	6 (1)
Asian	42 (0)	0 (0)
Black or African American	1,737 (14)	101 (16)
White	9,764 (79)	474 (76)
Age, N, mean (SD)	72.69 ± 9.70	72.74 ± 9.77
Patient Type, N (%)		
Dx of GCA as outpatient	11,435 (92)	555 (89)
BMI, mean (SD)	28.65 ± 5.80	29.01 ± 6.05
^a^Charlson, N, mean (SD)	11,403; 4.10 ± 2.74	612; 5.14 ± 2.96
Smoking, N (%)	3,466 (28)	166 (27)
Admissions, mean (SD)	1.43 ± 2.83	2.01 ± 3.23
Anticoagulation within 5 years, N (%)	277 (2)	15 (2)
^b^Thrombocytosis≥450, N (%)	101 (6)	12 (13)
Aspirin within 5 years, N (%)	469(4)	19 (3)
Diuretic use within 5 years, N (%)	766 (6)	40 (6)
Hypertension within 5 years, N (%)	9,320 (75)	479 (77)

Data are reported as mean ± SD or N with percentages (%).

BMI, body mass index; GCA, giant cell arteritis; SD, standard deviations.

a 1014 among patients without a VTE and 14 among patients with a VTE have missing data on Charlson score.

b 12316 among patients without a VTE and 614 among patients with a VTE diagnosis have missing data on thrombocytosis.

**Table 2:** Predictors for the incidence of PE after diagnosis of GCA.

Predictor	Univariable regression		Multivariable regression	
	Hazard ratio	p	Hazard ratio	p
Age	1.00 (0.99-1.02)	0.20	1.01 (0.99-1.02)	0.13
Black Race	1.27 (0.91-1.77)	0.15	1.30 (0.93-1.83)	0.12
Sex (F)	0.64 (0.35-1.18)	0.15	0.76 (0.41-1.41)	0.39
BMI	1.01 (0.99-1.03)	0.37	1.01 (0.98-1.03)	0.53
Smoking	0.84 (0.63-1.11)	0.22	0.80 (0.59-1.09)	0.17
Charlson comorbidity index	1.13 (1.09-1.18)	**<0.0001**	1.13 (1.08-1.18)	**<0.0001**
GCA dx outpatient vs. inpatient	0.65 (0.43-0.97)	**0.03**	0.78 (0.52-1.17)	0.23
Admissions	1.05 (1.02-1.08)	**0.0006**	1.02 (0.98-1.06)	0.21
Anticoagulation use within 5 y	0.80 (0.29-2.15)	0.65	0.61 (0.22-1.66)	0.33
Aspirin use within 5 y	0.78 (0.37-1.66)	0.53	0.63 (0.29-1.36)	0.24
Diuretic use within 5 y	1.27 (0.78-2.06)	0.32	1.17 (0.71-1.94)	0.53
Hypertension	1.11 (0.83-1.48)	0.46	0.93 (0.68-1.27)	0.67
Thrombocytosis	1.00 (0.24-4.17)	0.99	NA	NA

F, female; dx, diagnosis; y, year; NA, not selected by the multivariate model because of a large proportion of missing values, Bold values highlight the statistical significance of the results related to p-values.

**Table 3:** Predictors for the incidence of DVT after diagnosis of GCA.

Predictor	Univariable regression		Multivariable regression	
	Hazard ratio	p	Hazard ratio	p
Age	1.01 (1.00-1.02)	**0.02**	1.01 (1.00-1.02)	**0.03**
Black Race	1.09 (0.84-1.43)	0.48	1.08 (0.83-1.42)	0.55
Sex (F)	0.76 (0.49-1.17)	0.21	0.90 (0.58-1.40)	0.76
BMI	1.00 (0.98-1.02)	0.65	1.01 (0.99-1.02)	0.59
Smoking	0.95 (0.77-1.18)	0.69	0.95 (0.76-1.20)	0.74
Charlson comorbidity index	1.12 (1.09-1.16)	**<0.0001**	1.10 (1.06-1.14)	**<0.0001**
GCA dx outpatient vs. inpatient	0.55 (0.41-0.74)	**0.0001**	0.67 (0.50-0.91)	**0.01**
Admissions	1.06 (1.04-1.09)	**<0.0001**	1.04 (1.01-1.07)	**0.0004**
Anticoagulation use within 5 y	1.47 (0.83-2.61)	0.18	1.09 (0.59-2.01)	0.76
Aspirin use within 5 y	0.93 (0.55-1.6)	0.81	0.77 (0.44-1.34)	0.36
Diuretic use within 5 y	1.03 (0.69-1.54)	0.87	0.85 (0.56-1.30)	0.47
Hypertension	1.13 (0.91-1.41)	0.25	0.90 (0.71-1.13)	0.38
Thrombocytosis	3.09 (1.58-6.06)	**0.0009**	NA	NA

F, female; dx, diagnosis; y, year; NA, not selected by the multivariate model because of a large proportion of missing values, Bold values highlight the statistical significance of the results related to p-values.

Using the R package rms, nomograms were created for each type of thromboembolic events with covariates selected by Cox models. Calibration curves were then plotted to evaluate the calibration ability of our nomograms. Calibration was also based on cross validation.

Stratified Kaplan-Meier estimates of the outcomes are also presented in our study, where the grouping is based on the predicted risk score of multivariable Cox models. Subjects having a risk score higher (or lower) than the sample median were classified as high (or low) risk respectively. Two well- separated Kaplan-Meier plots would indicate a better predictive ability of the Cox models. The log-rank test was used to assess comparisons. Moreover, to quantify the predictive power of the multivariable Cox model, we also report the testing concordance index, which is calculated using 5-fold sample splitting (20). All statistical analyses were done in R (version 3.6.3, http://www.r-project.org/).

## Results

### Baseline demographic characteristics of our study population

A total of 13,029 patients with a diagnosis of GCA were included in our analysis. Among them, 626 were diagnosed with thromboembolic events (PE and/or DVT) after the diagnosis of GCA, whereas 12,403 were not. Specifically, 261 patients developed PE and 440 patients DVT, whereas 75 patients developed both PE and DVT after the diagnosis of GCA. Our stratified summary of baseline covariates is presented in **Table 1**. The majority patients were white male. The mean age of patients within our two study groups was above 72 years old.

The initial diagnosis of GCA was placed at outpatient visits in more than 80% of the patients and the mean BMI was approximately 29 for both study groups with and without thromboembolic events. The 5-year Charlson comorbidity index was slightly higher in the group with thromboembolic events reflecting a worse state of health, compared to the group without thromboembolic events. Less than one third of the patients with GCA in both groups were recorded as smokers. Admission data in **Table 1** shows that the mean number of hospital admissions for any cause was slightly higher in patients with GCA with thromboembolic events compared to patients without.

Only 2% of the patients in both groups were on anticoagulation treatment within 5 years prior to diagnosis of GCA. Interestingly, a higher percentage of patients with GCA that developed thromboembolic events had thrombocytosis, compared to patients without thromboembolic events. Similar percentages of both groups were on aspirin or diuretic therapy within 5 years prior to the diagnosis of GCA. The frequency of hypertension, which is an independent risk factor for thromboembolism (21), was similar in patients within both groups.

### Risk factors of thromboembolic events among patients with GCA

We explored potential predictors of developing PE and DVT during an observation period of five years after the diagnosis of GCA using Cox regression models (**Tables 2**, **3**). In the univariable Cox regression analysis, for the outcome of PE, the HR was 1.13 (p<0.0001) for Charlson comorbidity index, 1.05 (p=0.006) for hospital admissions, 0.65 (p=0.03) for diagnosis of GCA in the outpatient setting (left column, **Table 2**). In the multivariable analysis, only Charlson index (HR 1.13, p<0.0001) was significantly associated with the outcome of PE (right column, **Table 2**).

On the other hand, age (HR 1.01, p=0.02), Charlson comorbidity index (HR 1.12, p<0.0001), diagnosis of GCA in the inpatient setting, and hospital admissions before the diagnosis of GCA (HR 1.06, p<0.0001), were strong independent predictors of DVT in our multivariable model (right column, **Table 3**). Results were different for the univariable analysis, where thrombocytosis was also found to be a predictor of DVT (HR 3.09, p=0.0009), as depicted in the left column of **Table 3**. In conclusion, Charlson comorbidity index was a statistically significant independent risk factor for both PE and DVT. Other covariates may also have predictive ability as we show later in this study.

### Evaluation of predictive models

**Figure 1:**
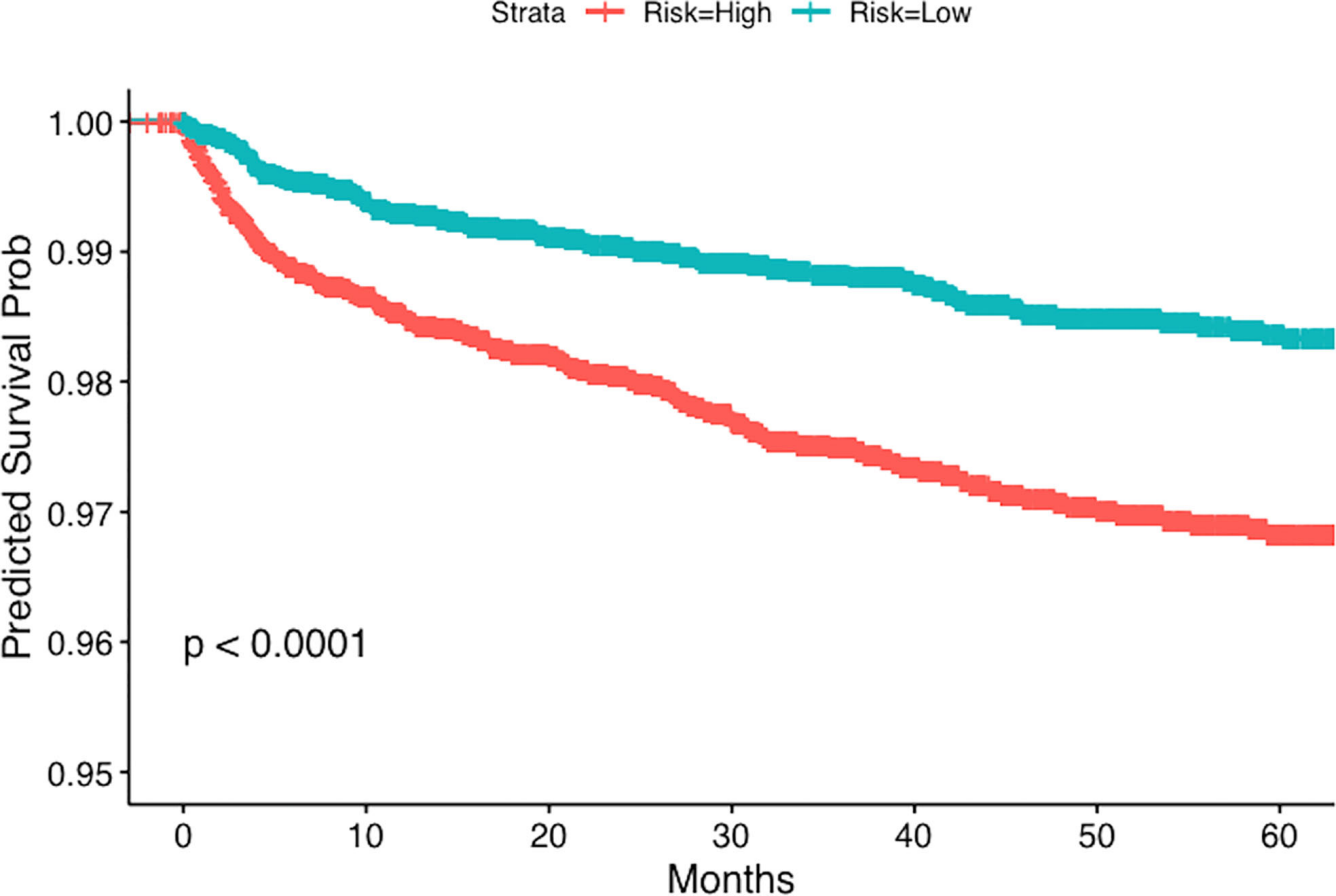
Kaplan-Meier plot for the time to PE from the time of diagnosis of GCA stratified by predicted risk (blue and red lines) respectively.

**Figure 2:**
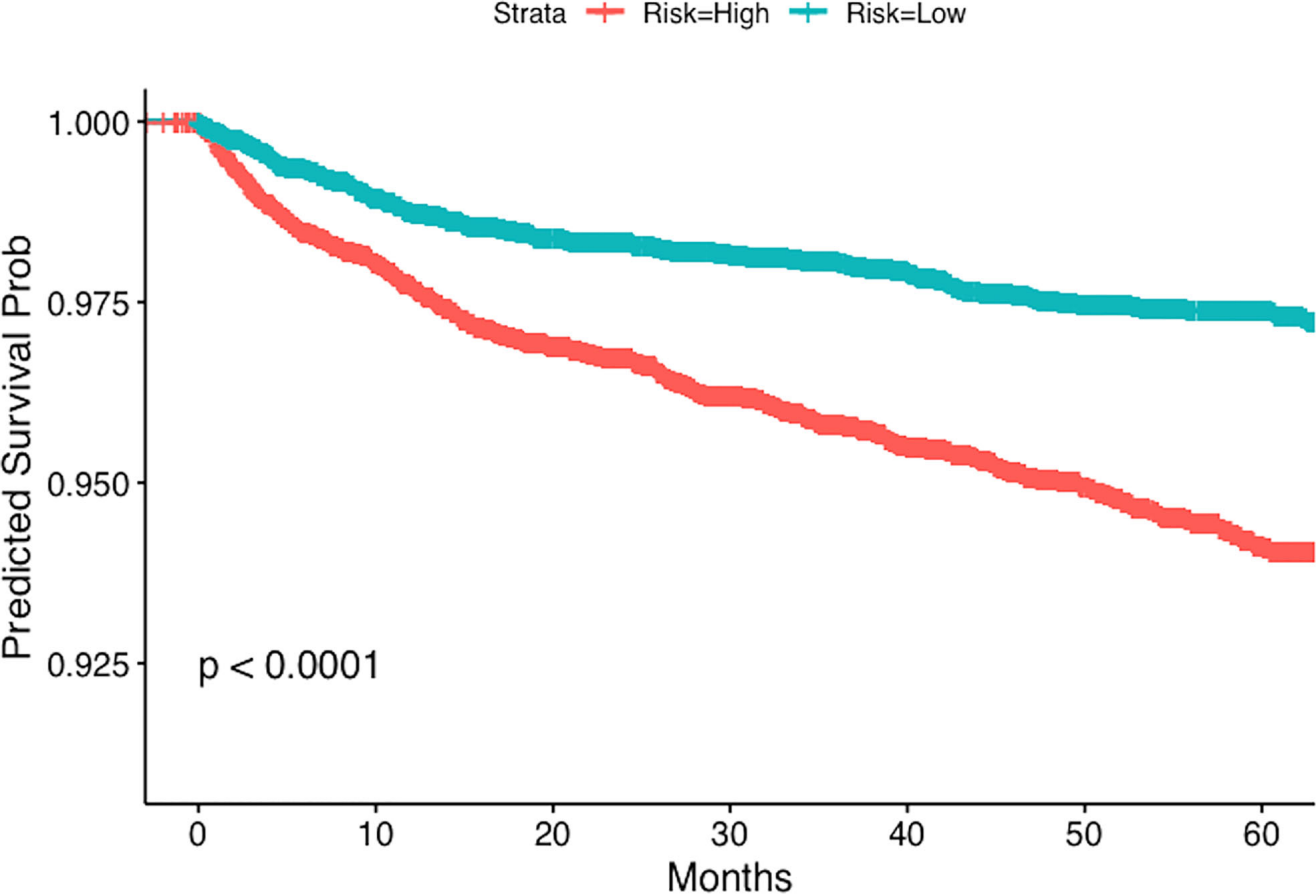
Kaplan-Meier plot for the time to DVT from the time of diagnosis of GCA stratified by predicted risk (blue and red lines) respectively.

We present the stratified Kaplan-Meier curves for outcomes of PE and DVT in **Figures 1**, **2** respectively. As demonstrated in these figures, patients with GCA, corresponding to a higher risk score (according to the multivariable Cox model), have distinct time-to-event incidence of PE or DVT events compared to those with a lower score. The x-axis represents time in months after the diagnosis of GCA and the y-axis depicts the probability of patients with GCA surviving without experiencing PE or DVT. The blue and red lines represent Kaplan-Meier plots of the low and high-risk groups of GCA for PE or DVT. For example, at 40 months after the diagnosis of GCA, the predicted probability of experiencing PE is almost 1% for the low-risk group, whereas the patients in the predicted high-risk group have approximately 2.5-fold greater chance of developing PE (**Figure 1**).

For PE the testing concordance index (C-index) is 0.62 ± 0.02, and for DVT it is 0.61 ± 0.01, indicating a good predictive discrimination. The standard error was estimated using bootstrap. These results suggest that if we know the baseline covariates (as listed in **Tables 2**, **3**) of two patients with GCA there is around 60% chance that we can correctly predict which one will develop PE or DVT events earlier, based on our multivariable regression models.

### Nomograms of thromboembolic events for patients with GCA

**Figure 3:**
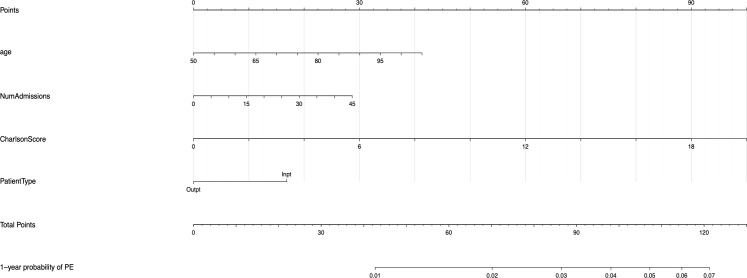
Nomogram for predicting the 1-year probability of PE in patients with GCA. NumAdmissions, number of admissions; Outpt, outpatient; Inpt, inpatient.

**Figure 4:**
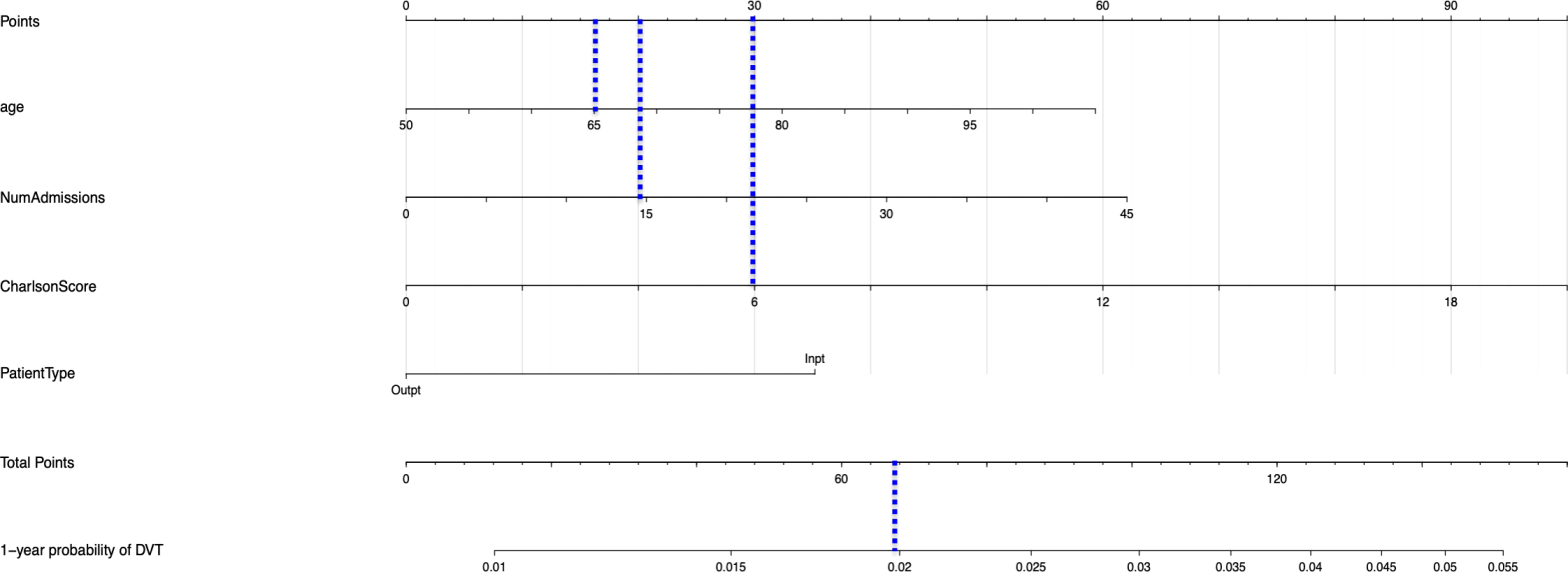
Nomogram for predicting the 1-year probability of DVT in patients with GCA. NumAdmissions, number of admissions; Outpt, outpatient; Inpt, inpatient.

**Figure 5:**
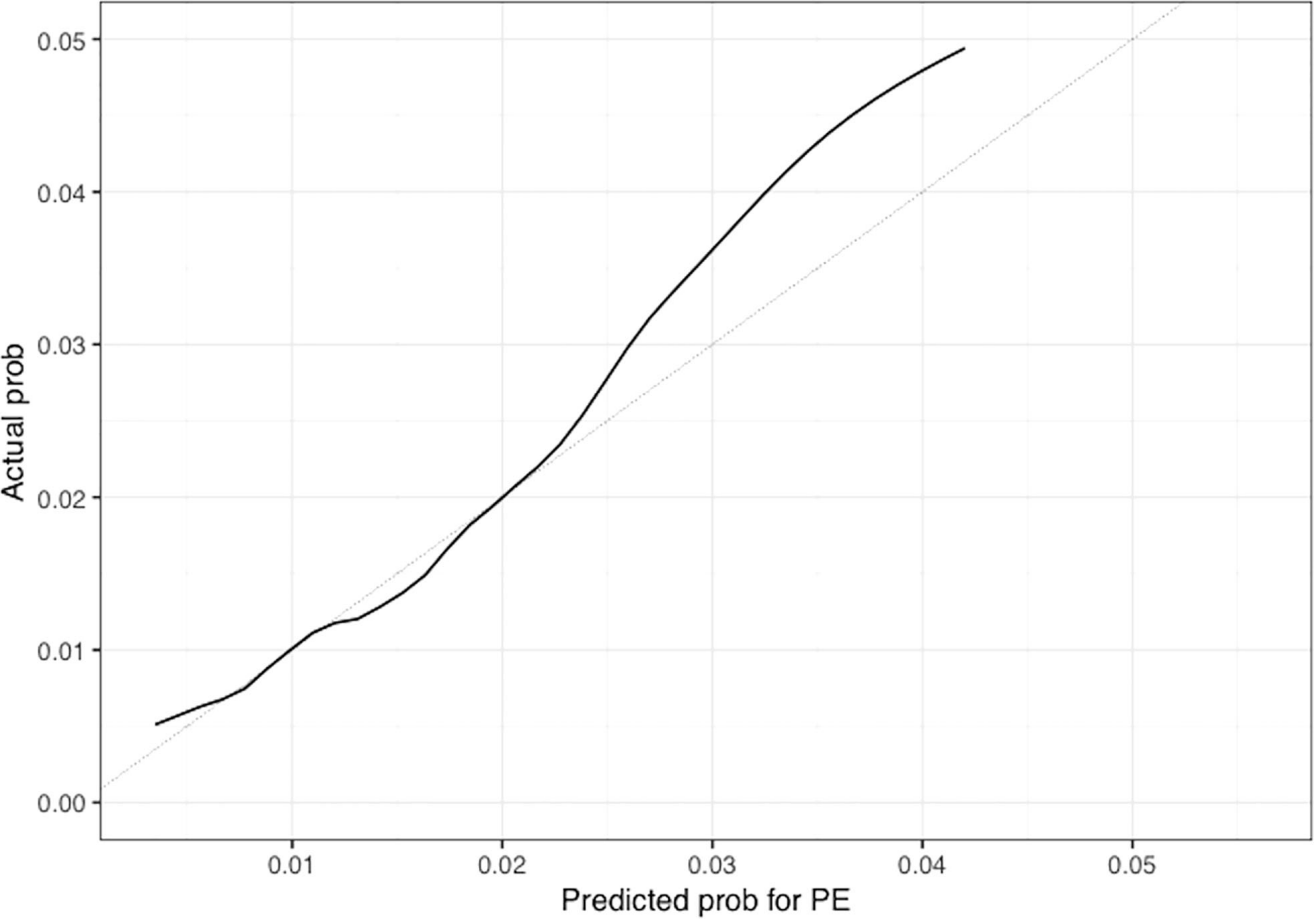
Calibration plot of the nomogram for predicting 1-year overall probability for PE in patients with GCA. Nomogram-predicted probability for PE is plotted on the x-axis while actual probability is plotted on the y-axis; The grey line represents the ideal line. .

**Figure 6:**
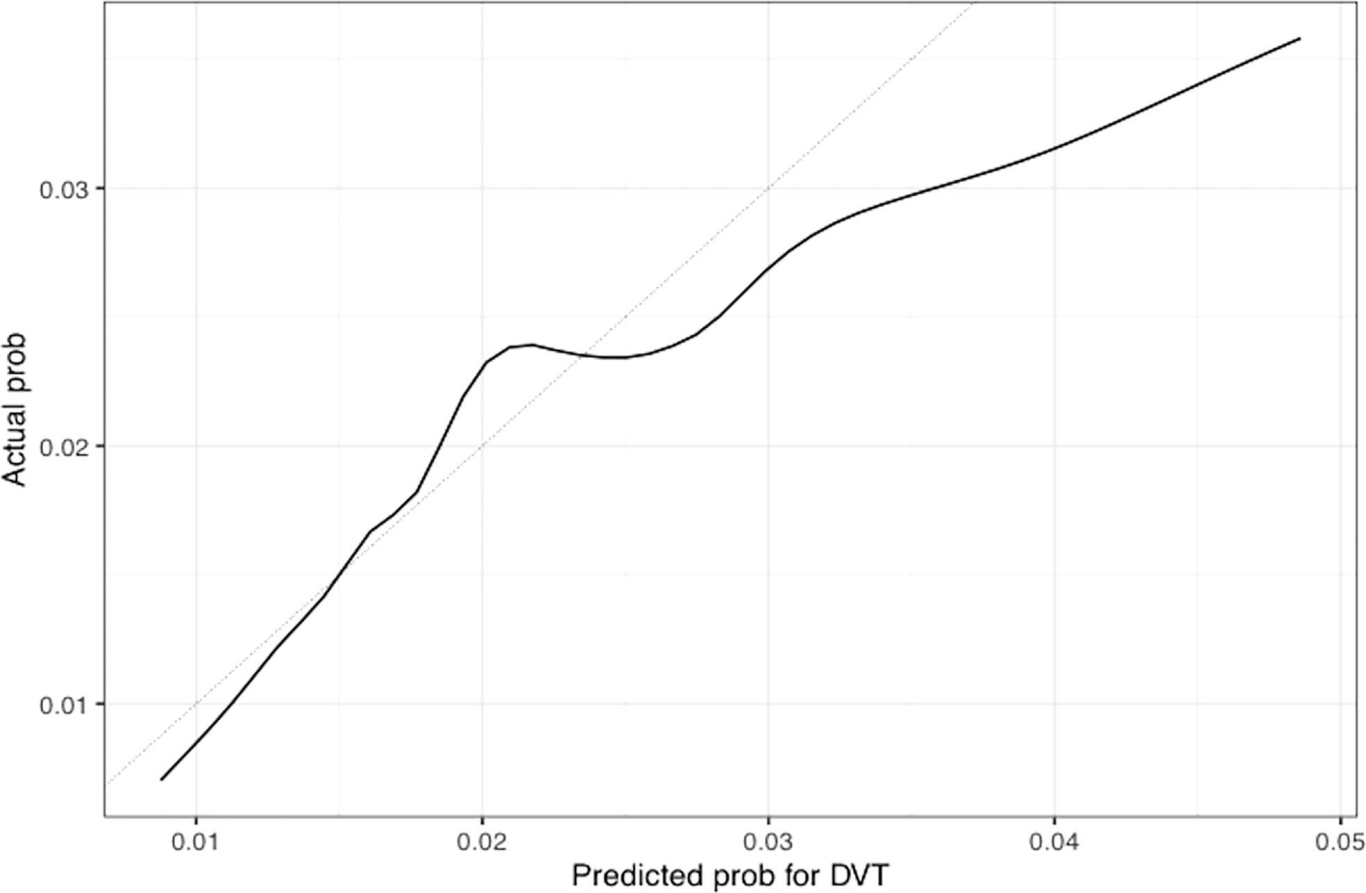
Calibration plot of the nomogram for predicting 1-year overall probability for DVT in patients with GCA. Nomogram-predicted probability for DVT is plotted on the x-axis while actual probability is plotted on the y-axis; The grey line represents the ideal line.

The univariable and multivariable Cox regression analyses indicate that age, Charlson comorbidity index, diagnosis of GCA in the inpatient setting, hospital admissions for any cause before the diagnosis of GCA, and thrombocytosis are independent factors that are associated with the occurrence of thromboembolic events in patients with GCA. However, these models do not give a simple or direct quantity that physicians can use readily in practice. For this purpose, we created nomograms based on some of these predictive variables to estimate the 1-year probability of developing PE or DVT in patients with GCA (**Figures 3**, **4**). We also present the calibration curves for the nomograms to evaluate their accuracy (**Figures 5**, **6**). For both PE and DVT outcomes, the predicted risk is in agreement with the apparent proportion for low to moderate risk patients, and this statistical regime includes most of the patients. However, for the PE event the nomogram would overestimate the risk for patients having a higher risk >0.03 that may be due to the linearity assumption of logistic regression. On the other hand, for the DVT event the nomogram works well for the whole range of risk.

To read the nomogram, for each variable draw a straight line upward to the axis labeled as Points at the same vertical position, and then calculate the total points. Afterwards, draw a straight line downward to the axis labeled as 1-year probability PE or DVT at the same vertical position to find the patient’s likelihood of 1-year probability of PE or DVT. For instance, a 65 year-old patient with Charlson comorbidity index of 6 is admitted to the hospital 15 times before the diagnosis of GCA. The points for each of the variables are 18 (65 year-old), 30 (Charlson score of 6), and 20 (15 hospital admissions before diagnosis of GCA); the patient has a total score of 68 points, and the 1-year probability of developing DVT turns out to be 0.02 (**Figure 4**).

## Discussion

In this retrospective study, we propose four independent predictive factors for thromboembolic events among veterans diagnosed with GCA that include age, Charlson comorbidity index, initial diagnosis of GCA in the inpatient setting, and number of hospital admissions before the diagnosis of GCA in our multivariable model. We then constructed a clinical prediction model based on these predictive factors to predict the 1-year thromboembolic event probability of patients with GCA at any time after the diagnosis of GCA, which could guide clinicians in their practice and advance the health of patients with GCA by creating thromboembolic risk stratification models. Future prospective multi-center studies are required to validate the accuracy and effectiveness of our prediction models/nomograms.

Most of the predictive factors for thromboembolic events that are proposed in this study are consistent with previous studies. Similar to our finding, Charlson comorbidity index has been identified as a predictor of VTE among high-risk cancer patients undergoing chemotherapy in the ambulatory setting (22). In the Khorana risk score, increased platelet count was also found to be an important predictor for VTE among patients with cancer (23, 24). Although thrombocytosis was significantly associated with the outcome of DVT, it was not included in our predictive models because of missing data, but its validity as a potential risk factor for VTE can be assessed in prospective multicenter studies. Older age has also been recognized as a predictor for VTE (25). In another study there was moderate evidence that there is a probable association between risk of VTE and increasing age (≥60 years old) in admitted medical patients (26).

Our finding that hospital admissions is a strong independent predictor of thromboembolic events among patients with GCA is supported by existing literature that shows that hospitalized GCA patients are at a greater risk of VTE (11). Our overall findings lay the foundation for future studies investigating the complexity of interactions between thrombo-inflammation, aging, multi-morbidities, and thromboembolic risk in patients with GCA, and/or other inflammatory conditions.

One of the limitations of our study is that potential confounders such as, sepsis, trauma or surgery, were unable to be taken into consideration, and residual confounding is a possibility. Another limitation is possible selection bias because of the retrospective nature of this study. It is also uncertain whether our results can be applied to the general population as the military population is represented heavily by men. It is well known in the literature that males with GCA are at higher risk of developing VTE compared to females with GCA (27). Finally, histologic evidence of temporal arteritis, representing the gold standard for the diagnosis of GCA, was not available for patients limiting diagnostic accuracy.

There is also a possibility of misclassification, as we did not include in the definition of GCA, glucocorticoid therapy for at least 6 months to avoid survival bias. For example, patients with GCA that developed fatal PE may not have survived for up to 6 months to receive corticosteroid therapy for the full period. Thus, glucocorticoid therapy was not part of our inclusion criteria, as our main goal was to identify high-risk patients at the time of GCA diagnosis. Additionally, inflammatory markers, clinical subtypes of GCA, advanced imaging studies such as magnetic resonance angiography and fluorodeoxyglucose (FDG)-positron emission tomography, and disease activity status, were not recorded for this study.

Our study has several strengths. This is the first large study that proposes independent predictors and risk model for thromboembolic events among patients with GCA diagnosis in a US military population. Our findings may help physicians to accurately calculate with readily available clinical risk factors the probability of developing PE or DVT among veterans with GCA over a period of one year, integrating specific predictive variables that can easily be extracted from the medical chart for most hospitals and academic centers, by using our nomogram model as a reference.

The results of our study may also assist physicians to stratify patients with GCA at low and high risk for VTE and predict the probability of a thromboembolic event over an observation period of five years in a male population with GCA. Additionally, this stratification strategy may be useful for developing strategies for screening of lower extremities with Doppler ultrasonography among high-risk GCA patients for VTE in an attempt to identify asymptomatic events of DVT (28, 29).

In conclusion, use of thrombosis predictive models to predict the risk of VTE may help with risk stratification of patients at increased risk according to the probability of a thromboembolic event. Thus, there is an unmet need for further validation of the clinical applicability of these predictive models by large multicenter, prospective clinical studies.

## Data availability statement

The datasets presented in this article are not readily available due to ethical/privacy restrictions. Requests to access the datasets should be directed to BN, Bernard.Ng@va.gov.

## Ethics statement

The Research and Development Committee from the Department of Veterans Affairs approved the study (MIRB#01854). Written informed consent for participation was not required for this study in accordance with the national legislation and the institutional requirements.

## Author contributions

DM, TZ, NK, GL, AD, PS, and BN contributed to the conception and design of this study. DM, TZ, and BN participated in data collection and data analysis. Only DM and BN had access to raw data. All authors contributed to data interpretation, critically reviewed and revised the manuscript and approved the final submission.

## Funding

DM is supported by the T32 NIH grant from Nutrition, Obesity and Atherosclerosis (#5T32HL007028-44). The funder body was not involved in the study design, collection, analysis and interpretation of the data, the writing of this article or the decision to submit it for publication.

## Conflict of interest

DM received Advisory Board fees from ChemoCentryx and a research grant from Pfizer US pharmaceuticals. NK reports consulting fees from Beyond Spring, Bristol Myers Squibb, G1 Therapeutics, Sandoz, Seattle Genetics, Janssen, Pfizer, and Spectrum and research funding from Amgen to her institution (family member). GL reports research grant support from Amgen to the Fred Hutchinson Cancer Center and consulting fees from Beyond Spring, G1 Therapeutics, Partner Therapeutics, Samsung Bioepis, Merck, Jazz, TEVA, Squibb, Sandoz, Seattle Genetics, and Fresenius Kabi.

The remaining authors declare that the research was conducted in the absence of any commercial or financial relationships that could be construed as a potential conflict of interest.

## Publisher’s note

All claims expressed in this article are solely those of the authors and do not necessarily represent those of their affiliated organizations, or those of the publisher, the editors and the reviewers. Any product that may be evaluated in this article, or claim that may be made by its manufacturer, is not guaranteed or endorsed by the publisher.
