# Supplementary material for: Predictive models for thromboembolic events in giant cell arteritis: A US veterans health administration population-based study
Source: Front Immunol. 2022 Nov 9;13:997347. doi: 10.3389/fimmu.2022.997347 (PMC9681825; doi:10.3389/fimmu.2022.997347)
Supplement: Supplementary file 1 [file Table_1.docx]

**Supplementary Table 1. ICD-9 and ICD-10 CM of diseases of interest**

| **Diagnosis** | **ICD-9 CM** | **ICD-10 CM** |
| --- | --- | --- |
| GCA | 446.5 | M31.6 |
| PE | 415.xx | I26.xx |
| DVT | 453.xx | I82.4xx, I82.629 |
| Hypertension | 401.9 | I10 |
| Exclusive diagnosis (malignancy) | 199, 199.1, 415.11 | C80.11 |

GCA, giant cell arteritis; PE, pulmonary embolism; DVT, deep venous thrombosis;, ICD-9 CM, International classification of diseases Ninth Revision clinical modification code; ICD-10 CM, International classification of diseases Tenth Revision clinical modification code
